# Exploring preparatory reading in bidirectional sight and written translation through clustering analysis of eye-tracking data

**DOI:** 10.1371/journal.pone.0329858

**Published:** 2025-08-26

**Authors:** Shirong Chen, Jia Feng, Michael Carl

**Affiliations:** 1 School of International Studies, University of International Business and Economics, Beijing, China; 2 School of Foreign Languages, Renmin University of China, Beijing, China; 3 Department of Modern & Classical Language Studies, Kent State University, Kent, Ohio State, United States of America; Universiti Sains Malaysia, MALAYSIA

## Abstract

Preparatory reading—the phase between a translator’s initial reading of the source text and the production of the first word of the target text—remains underexplored despite its crucial role in both sight (SiT) and written translation (WT). This study examined preparatory reading patterns of 32 student translators, focusing on the effects of translation mode (SiT vs. WT) and direction (L1-to-L2 vs. L2-to-L1). Translators’ attention allocation, cognitive effort, and reading speed were measured using preparatory reading duration, average fixation duration, and fixation rate (i.e., number of fixations per second) as key indicators. Using linear mixed-effect models, we quantified the effects of translation mode and direction on each measure. We then synthesized all three measures by employing unsupervised machine learning algorithms (i.e. *k*-means cluster analysis) to identify distinct reading patterns. These quantitative findings were further complemented by expert qualitative categorization of reading patterns, achieved through subjective coding of scanpaths and Translation Process Graphs. We found that translation mode played a primary role in shaping preparatory reading styles, with SiT consistently requiring greater cognitive effort, more attention, and slower reading speeds than WT—particularly under the L1-to-L2 condition. Translation direction further modulated these effects in nuanced ways. Specifically, L2-to-L1 was associated with increased attention allocation in WT, but with less cognitive effort and faster reading in SiT. Additionally, we found three distinct preparatory reading patterns: *Fast Surface-level Preparatory Reading, Systematic Deep-level Preparatory Reading,* and *Extended Iterative Preparatory Reading,* each reflecting a distinct combination of cognitive investment and reading speed. These findings could advance our understanding of translators’ preparatory reading behaviors and underscore the need to equip them with adaptable, task-sensitive reading strategies that align with the cognitive demands of different translation modes and directions.

## Introduction

As part of descriptive translation studies, cognitive translation and interpreting studies (CTIS) regards the description and characterization of translators’ behavioral patterns as one of its fundamental goals. This, in turn, facilitates the empirical modeling of cognitive processes in translation and interpreting (T&I), helping to explain and predict the underlying cognitive mechanisms of translators and interpreters [1]. To achieve this objective, researchers have applied eye-tracking technology—valued for its high spatial and temporal resolution—to explore translators’ reading behaviors in a fine-grained way.

Eye-tracking investigations into translators’ reading behaviors have primarily focused on written translation (WT) [2–6]. Researchers have explored translators’ reading patterns across different phases of WT, including a) preparatory reading of source texts during the orientation phase, b) coordinated reading and writing behaviors during the drafting phase, and c) reading for revising target texts during the revision phase. Recently, reading patterns in less-researched modes of translation, particularly sight translation (SiT) — defined as “the oral translation of a written text” [7] — have attracted considerable interest. Sight translators’ reading behaviors and their coordination between reading and production were among the first behavioral patterns being explored [8–13].

In addition to profiling translators’ behavioral patterns within a single translation mode, recent studies have begun to compare different translation modes. These efforts aim to provide a more nuanced understanding of the underlying cognitive processes for each mode. As “a hybrid form of translation and interpreting” [14], SiT became a natural straddling point to start such cross-mode comparison. SiT has often been compared with simultaneous interpreting (SI) and consecutive interpreting (CI), with a variety of topics such as source text inference [15], information retention [16], and interpreting performance [17,18].

However, only a limited number of studies have focused on the comparison between SiT and WT [19,20]. Despite the limited research, recent years have seen growing calls for greater synergy between SiT and WT, both professionally and pedagogically. Professionally, an increasing number of translators are incorporating automatic speech recognition (ASR) technologies into WT workflows. Benefits of this synergy include streamlining workflows to increase efficiency, reducing typing-induced technical effort during WT, and minimizing the risk of repetitive strain injuries [21], all without compromising the translation quality [7,20]. Pedagogically, training programs are increasingly adapting to these changes by integrating SiT skills into the traditional WT curriculum, reflecting a growing recognition of this synergy’s role in enhancing translator proficiency and versatility. This trend toward greater synergy of the two modes underscores the importance of understanding the commonalities and differences in translators’ behavioral patterns across SiT and WT. Yet there remains much to be explored.

A fundamental step shared by both SiT and WT is the preparatory reading process, where translators pre-read the source text before producing the first word of the target text, through speaking or typing [17,22]. Against this backdrop, we conducted an eye-tracking study on bidirectional SiT and WT, investigating translators’ reading patterns during preparatory reading and exploring the potential influence of translation mode and direction. The study aims to provide a more nuanced understanding of preparatory reading behaviors in bidirectional SiT and WT and to uncover the possible interplay between translation mode and direction in modulating cognitive processing of translation.

### Sight translation as a special mode of translation and interpreting

Owing to its hybrid features, SiT lends itself to comparative research, offering insights into the shared and unique characteristics of various T&I modes. One strand of such research on SiT has adopted a product-oriented perspective, comparing SiT with other modes of interpreting. Agrifoglio [17] compared the performance of prepared SiT with consecutive interpreting (CI) and simultaneous interpreting (SI) and found that the continuous presence of the source text in SiT hindered the efficient production of target texts. However, Lambert [18] reported contrasting results, observing that the presence of source text could enhance interpreting performance in SiT, compared to SI and simultaneous interpreting with text (SI-Text). These conflicting results may be related to the differences in their preparatory reading conditions. Specifically, Lambert [18] allowed translators 10 minutes to prepare before sight translating a 7-minute speech segment, whereas Agrifoglio [17] provided less than five minutes for a 9-minute speech. Since “time and resources allowed for preparation” is one of the critical factors influencing the perceived difficulty of interpreting [23], it is reasonable to postulate that variations in preparatory reading may lead to different cognitive processing and interpreting performance. Nevertheless, the role of preparatory reading in translation, particularly in SiT, remains largely underexplored.

Another strand of empirical research compared SiT with WT in terms of cognitive processing, reflecting the growing advocacy for a greater synergy between SiT and WT [7]. Jiménez Ivars [24] found that the two modes involved different types of problems and strategies, with SiT requiring deeper cognitive processing and placing higher demands on the psycho-physiological components of translation competence than WT. Shreve et al. [25] compared SiT with WT in terms of the effects of syntactic complexity on cognitive effort, and found that SiT was more sensitive to syntactic disruptions than WT. While these studies offered valuable insights into the shared and divergent cognitive processes of SiT and WT, their findings remain preliminary and general. A more detailed, phase-level comparison of SiT and WT warrants further investigation. For instance, examining how translators pre-read the source text during the orientation phase could reveal their strategic adjustments for navigating the cognitive demands of each mode, thereby offering valuable insights into underlying cognitive processes.

### Preparatory reading in sight translation and written translation

Preparatory reading is an integral and crucial step of translation, referring to the process in which translators read the source text to familiarize themselves with its content and context before beginning their translation.

Investigations into preparatory reading patterns started from WT. As early as 1986, Krings [26] identified two WT preparatory reading patterns drawing on translators’ think-aloud protocols. *The successive processing strategy* involves reading the entire text for global comprehension. *The pre-translation relief strategy* involves reading the source text to fully understand its details and resolve potential problems. Later research by Shreve et al. [27] further uncovered a wider range of WT preparatory reading patterns: *skimming*, where translators read only headings and titles; *selected reading,* which involves the reading of specific sections or segments; and *serial reading*, wherein translators read and translate an entire textual segment before moving on to the next segment.

More recently, a more detailed understanding of WT preparatory reading patterns has emerged through integrative analysis of eye-movement data and key-logging data [2,3,28]. In particular, translation progression graphs (TPGs), i.e., fine-grained visualizations of how reading and production activities unfold and coordinate over time and space, were used to elucidate translators’ behavioral patterns. Four preparatory reading patterns were identified from the observations and categorizations of TPGs: a) *head-starter*, i.e., initiating translating immediately without pre-reading the source text, b) *quick-planner*, i.e., reading the first few words or sentences of the source text before translating; c) *scanner*, i.e., scanning the source text quickly before translating, and d) *systematic-planner*, i.e., reading through the source text systematically before translating.

Building on this seminal classification, Dragsted and Carl [2] found that, under time pressure, most translators were head-starters, followed by quick-planners and scanners, with systematic-planners being the least prevalent. However, an opposite trend was reported by Feng [28], who found that without time constraints, most student translators were systematic planners, followed by quick-planners and scanners, with head-starters being the least frequent. Additionally, translation direction appeared to influence the distribution of patterns: more systematic planners were identified in L2-to-L1 direction, whereas L1-to-L2 direction involved relatively more scanners, quick-planners, and head-starters. These preliminary findings, based on qualitative coding of TPGs, highlight the complexity and diversity of preparatory reading patterns and underscore the moderating roles of factors such as time pressure and translation direction.

Compared to WT, SiT preparatory reading has received relatively less focused scholarly attention. Among the earliest explorations, Mikkelson [29] proposed a sequential macro-micro model from a prescriptive point of view. In the initial *macro-reading*, the translator scans the source text to understand its meaning rather than pondering on how to translate specific terms. In the subsequent *micro-reading*, the translator inspects the text in detail. Moving beyond this broad theoretical proposition, a limited number of empirical studies have provided further insights. Lee [30] found that professional interpreters read faster and more efficiently than students during SiT preparatory reading. This inefficiency among students was attributed to their reliance on *bottom-up strategies* in reading, focusing on decoding or concentrating on individual words or phrases. In contrast, more efficient readers employed *top-down strategies*, emphasizing conceptual understanding and global meaning. Moreover, efficient reading patterns also tended to combine top-down and bottom-up strategies.

Recent applications of eye-tracking technology have enabled finer-grained exploration of reading patterns. One of the earliest pieces of eye-tracking evidence about SiT preparatory reading came from Chmiel and Mazur [31], who invited two groups of translators (advanced vs. less advanced students) to pre-read the first page of a 345-word source text within 10 seconds. The assumption was that the more advanced group would engage in “*gist reading*” to gather maximal information quickly, while beginners tend to engage in “*linear reading*”, processing from the beginning of the text. Gist reading was expected to result in more extensive scanning and thus more fixations. However, this expectation was not confirmed. Instead, less advanced students were found to fixate more frequently and scan a wider range of text, though these differences were not statistically significant. Later, Su and Li [12] used eye-tracking to examine self-paced Chinese-English SiT preparatory reading among 14 student translators. On average, translators spent 63.69 seconds pre-reading a 50-word source text. Scanpath visualizations suggested two general SiT preparatory reading patterns: *linear readers,* who demonstrated less reinspective fixations, and *local readers*, who engaged in more re-reading. Moreover, L1-to-L2 SiT preparatory reading was dominated by local readers, requiring translators more attention (total preparatory reading duration: 77.05 vs. 50.53 seconds), greater cognitive effort (average fixation duration: 262.11 vs. 236.09 milliseconds), and increased reprocessing effort (average reinspective fixation duration: 276.98 vs. 251.02 milliseconds). In contrast, L2-to-L1 SiT preparatory reading was dominated by linear readers.

Taken together, a review of literature suggests that preparatory reading patterns of SiT and WT exhibit both commonalities and distinctions. However, empirical studies comparing SiT and WT in terms of preparatory reading are notably scarce, despite the increasing call for greater synergy between the two modes. One existing study [32] offered preliminary results by examining the translation processes of 60 trainee translators, revealing that SiT preparatory reading involved significantly longer time than WT preparatory reading on average (172.5 vs 60.7 seconds).

### Research gaps and research questions

The preceding literature review underscores the important role of preparatory reading in both SiT and WT translation processes and highlights its potential impact on translators’ cognitive processing and translation products. Despite its importance, research on preparatory reading, particularly comparative studies between SiT and WT, remains limited. The limited body of existing research has revealed that translators’ preparatory reading behaviors are diverse and complex, and may be influenced by factors such as translation direction [2,12,28] and translation mode [32]. However, these findings remain limited and preliminary, requiring more targeted, robust, and finer-grained evidence from eye-tracking. Furthermore, the potential interactions between influencing factors such as translation mode and direction have yet to be fully explored.

Existing eye-tracking studies on preparatory reading have followed two main research orientations. The first, predominantly quantitative, focused on measuring the attention and effort invested in preparatory reading by quantifying eye-movement metrics, such as average fixation duration and fixation rate [30,31]. The second, more qualitative in nature, involved researchers observing and classifying visualizations of translators’ scanpaths [12,33] to identify distinct preparatory reading patterns. This qualitative approach, however, requires a thorough prior understanding of translators’ preparatory patterns, relies heavily on human judgment, and demands substantial manual effort, making it challenging to apply to large-scale data.

To address these challenges, this study introduced *k*-means cluster analysis to identify preparatory reading patterns, which, to the best of our knowledge, is the first application of this method in translation studies. Cluster analysis is widely used in related fields such as second language studies, where it is recognized as one of the most commonly used unsupervised machine learning algorithms [34]. By identifying natural groupings in the data, cluster analysis provides a clear and objective structure that can complement and enhance human subjective observations and classifications of translators’ preparatory reading patterns.

Against these backdrops, we conducted an eye-tracking study to investigate reading patterns during bidirectional SiT and WT preparatory reading, and to explore the extent to which translation mode (SiT vs. WT) and translation direction (L1-to-L2 vs. L2-to-L1) influence preparatory reading behavior. Two research questions (RQs) are raised:

RQ1: To what extent are translators’ preparatory reading behaviors modulated by translation mode and translation direction?

RQ2: What preparatory reading patterns can be identified in translators’ preparatory reading, and to what extent the preparatory reading patterns differ across two translation modes and two translation directions?

For both RQs, we examined three measures: a) preparatory reading duration, i.e., the time interval between the reading of the first source text word and the speaking or typing of the first target text word, b) average fixation duration, i.e., the mean duration of all fixations during preparatory reading, c) fixation rate, i.e., the number of fixations per second during preparatory reading. Specifically, preparatory reading duration reflects translators’ allocation of attention [10,12,28], with longer duration indicating a greater investment of attentional resources. Average fixation duration is a widely recognized indicator of cognitive effort in both cognitive translation studies and reading research [10,28,35,36]. Longer average fixations are consistently associated with increased processing demands—such as resolving linguistic complexity or unfamiliar content—and thus reflect more effortful cognitive processing [37]. Fixation rate reflects both attention speed [38,39] and reading speed [37,40], with a higher fixation rate indicating that more fixations occur per unit of time, thereby suggesting a faster speed of attention.

### The present study

#### Experiment design.

We conducted a quasi-experiment in 2021 at the eye-tracking laboratory of Renmin University of China, with ethical approval obtained (Approval No: 20210001). This experiment employed a 2 × 2 within-subjects design, with translation mode (i.e., SiT vs. WT) and translation direction (i.e., L2-to-L1 translation vs. L1-to-L2 translation) as two independent variables. Each participant was required to complete four translation tasks, including L1-to-L2 SiT, L2-to-L1 SiT, L1-to-L2 WT, and L2-to-L1 WT. The order of the translation tasks was counterbalanced using a Latin square design.

#### Source texts.

Two English and two Chinese texts were used as source texts for the SiT and WT tasks. The four texts were controlled for genre, key linguistic features, and translation difficulty. To ensure comparability of the four texts and suitability for our participants, we followed three main steps in preparing the experimental texts.

First, we selected four English and four Chinese news reports from major mainstream media outlets and compiled a preliminary pool of candidate texts. All news texts were comparable in linguistic features, including word count, word frequency, and readability level. Then, five experienced T&I trainers were invited to evaluate the difficulty of the eight texts for both SiT and WT tasks using a Likert Scale ranging from 0 (least difficult) to 10 (most difficult). In the third step, eight student translators with backgrounds comparable to those of our participants performed either SiT or WT using the eight source texts. After completing each task, they assessed the translation difficulty for each text on a 0–10 Likert scale. The translation difficulty for each text was determined by averaging the ratings from both the T&I trainers and the student translators. Based on these combined objective and subjective measures, two English and two Chinese texts were selected for their comparability in both linguistic characteristics and translation difficulty (see Table 1). In summary, the four experimental texts were selected to ensure that they were of intermediate difficulty and comparable both within and across languages.

**Table 1:** Text characteristics and translation difficulty of source texts.

Source text	Word count	Proportion of high-frequency words	Readability (CEFR level)	Translation difficulty by experts	Translation difficulty by students
English Text1	142	88%	B1	4.35	4.84
English Text2	143	92%	B1	4.80	4.77
Chinese Text1	152	92%	B1	5.15	4.47
Chinese Text2	148	89%	B2	6.40	4.22

Notes. 1. English and Chinese word frequencies were measured by COCA (Corpus of Contemporary American English) and LCMC (The Lancaster Corpus of Mandarin Chinese), respectively.

2. English and Chinese readability coefficients were first measured by Flesch-Kincaid grade level and HSK grade level (*Hanyu Shuiping Kaoshi*, i.e., Chinese Proficiency Test), respectively. The Flesch-Kincaid readability scores for the two English source texts were about 70, corresponding to a seventh-grade level, which aligns with the B1 level of the Common European Framework of Reference for Languages (CEFR). The HSK readability grade levels for Chinese source texts were about Middle 3 and 4, corresponding to the CEFR B1 and B2 levels.

### Participants

Participants in this study were 32 student translators, aged between 20 and 26 (*M =* 23.25, *SD =* 1.67). Student translators were deliberately selected to ensure comparable training backgrounds and eliminate variability from professional experience, thereby allowing the study to yield insights relevant to pedagogical practices in translator training.

Student translators were selected through convenience sampling from several high-ranking universities in China. They were included if they: a) had Chinese as their first language (L1) and English as their second language (L2); b) were majoring in translation or interpreting with formal university-level training in both WT and SiT; c) had completed at least one semester of formal training in both WT and SiT; and d) could touch type and had normal or corrected-to-normal vision. Only participants who met all these criteria were included in the study.

Eligible participants self-rated their translation proficiency in SiT and WT using a Likert scale ranging from 0 (no proficiency) to 10 (maximum proficiency). The average self-reported proficiency scores were 6.59 (*SD =* 1.41) for WT, 5.62 (*SD =* 1.39) for SiT, 6.44 (*SD =* 1.34) for L2-to-L1 translation, and 5.84 (*SD =* 1.39) for L1-to-L2 translation.

### Data collection

Translators’ eye movements were recorded by a remote eye-tracker (Tobii TX-300) with a sampling rate of 300 Hz. The eye-tracker was equipped with a 23-inch display screen with a resolution of 1920 × 1080 pixels. During the experiment, participants were seated in a fixed chair approximately 65 cm from the eye-tracker screen. Before each translation task, participants completed a 9-point eye-movement calibration to ensure the quality of the eye movement data.

**Fig 1:**
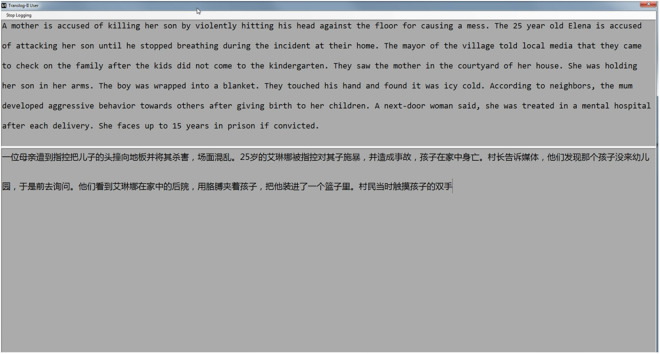
A demo of English-to-Chinese written translation interface.

For both SiT and WT tasks, Translog-II was used as the translation interface [41]. In the upper half of Translog-II, we presented the source text for each translation task (see Fig 1), while in the lower half, participants typed their target texts during WT tasks. In SiT tasks, participants’ spoken output was simultaneously recorded using Audacity 2.4.2. Regarding the typography, monospaced fonts were used: Consolas for English texts and Microsoft Yahei for Chinese texts [42]. A font size of 18 points and double spacing were consistently applied to all texts. Additionally, to alleviate participants’ eye strain and enhance their comfort during task, we configured Translog-II with a light-gray background.

Each participant performed two SiT tasks and two WT tasks without consulting any online or offline resources. They were instructed to work at their usual pace, including setting their own pace for the preparatory reading of the source text. Participants could end the task when they deemed that their target texts had reached their usual standards of satisfactory quality. Immediately after completing each translation task, participants filled out the NASA Task Load Index [43,44] to report their perceived overall cognitive workload of each translation task. Participants then took a mandatory 5-minute break before proceeding to the next task. After completing all four translation tasks, participants took part in a semi-structured interview, answering questions about how they allocated time, pre-read source texts, and produced target texts across two translation modes and directions. The aim of the interview was to elucidate the motives and rationale behind specific translation approaches and behavioral patterns. Moreover, as this study focused on cognitive processes during the preparatory reading phase, translation performance was not assessed. While we acknowledge its potential relevance, performance evaluation is beyond the scope of this process-oriented design.

### Eye-movement data cleaning

Ensuring the quality of eye-movement data is a critical issue in eye-tracking research, as noisy data increases the risk of distorted results. Following the data cleaning guidelines recommended by Eskenazi [45], we conducted initial pre-processing of the eye-movement data using Tobii Studio (version 3.4.7). The fixation filter was set to I-VT, with a minimum fixation duration threshold set at 60 ms. The first step in data cleaning involved excluding translation tasks with great data loss by examining the gaze sample value (GS, i.e., proportion of gaze samples captured by the eye tracker compared to the theoretical maximum) for each task. Drawing on previous translation studies [28,43,46], we excluded tasks whose GS values fell one standard deviation below the grand mean (*M =* 78.023%, *SD =* 14.413%), resulting in a threshold of 63.612%. Consequently, 16 tasks with GS values below this threshold were excluded from a total of 128 tasks. The average GS value of the remaining 112 sessions was 82.295% (*SD = *8.405%).

The second step involved removing fixations located outside the area of interest (AOI), defined as the source text region in Translog-II. The preparatory reading period—or orientation phase—defined as the time of interest (TOI), consisted of 35,106 fixations from 110 SiT/WT sessions, with two sessions excluded due to the absence of preparatory reading. A total of 182 fixations were found located outside the source text area and subsequently removed, accounting for 0.518% of all fixations. The third step involved excluding fixations shorter than 80 ms or longer than 800 ms, as fixations outside this range were considered atypical for cognitive processing during reading [47]. This resulted in the exclusion of 1,659 fixations, accounting for 4.750% of the remaining fixations. After three steps of data cleaning, the final eye movement dataset consisted of 33,256 fixations from a total of 110 translation tasks.

### Data analysis

The data analyses were conducted using R [48]. To answer the first research question, we built three linear mixed-effect models (LMMs), one for each of the three dependent variables—preparatory reading duration, average fixation duration, and fixation rate— using the *lme4* package [49]. *P-values* were obtained from the *lmerTest* package [50]. In each LMM, translation mode (Mode) and translation direction (Dir) were specified as fixed factors, while participants (Part) and source texts (Text) were included as random factors. Log transformations were applied to dependent variables to mitigate the negative impact of potential skewness.

When fitting the models, we began with a maximal random-factor structure that included all possible random intercepts and slopes, i.e., (Dir*Mode|Part)+(Mode|Text). However, when the full models failed to converge, we reduced the random-effects structure. Specifically, we first removed participant-level correlations and interactions between fixed factors. Upon successful convergence, we followed the “model selection approach” to further simplify the remaining random structure, aiming for a more parsimonious model [51]. This involved constructing a simpler model by excluding random components with near-zero variance and subsequently comparing it to the previous model using likelihood ratio tests. We assessed the normality of the residuals of the final parsimonious model by examining their kurtosis, skewness and quantile–quantile plots. If the residuals violated the normality assumption, data points beyond three standard deviations from the mean were excluded, and a refined model was constructed [45].

To answer the second research question, we conducted a *k*-means cluster analysis using the *factoextra* package in R [52,53]. *K*-means is a widely used unsupervised learning algorithm in second language research, effective for identifying latent learner profiles and behavior patterns [34]. It partitions data by minimizing within-cluster variance, iteratively assigning datapoints based on their proximity to cluster centroids in a multidimensional space. This method was chosen for its ability to detect distinct behavioral patterns from multidimensional eye-tracking data [54], and to generate non-overlapping clusters that capture variation in reading styles.

All three eye-tracking measures were first standardized to ensure comparability. The optimal number of clusters was determined using the “elbow” method, which plots the within-cluster sum of squares against the number of clusters to identify the inflection point balancing model simplicity and explanatory power [34]. To assess the robustness of the three-cluster solution, we computed silhouette coefficients (ranging from –1 to 1), which quantify how well each observation fits within its assigned cluster relative to others. Cluster-level silhouette scores indicated that Cluster 1 (0.59) and Cluster 3 (0.45) were well differentiated, while Cluster 2 (0.06) showed limited cohesion. Nonetheless, Cluster 2 was retained based on its distinct interpretive value and consistent qualitative patterns observed in the translation progression graphs and scanpath visualizations. To statistically examine the distinctiveness of the resulting clusters, we performed a one-way ANOVA to examine the potential differences among the identified clusters concerning the three indicators. Additionally, Fisher’s exact test was used to evaluate whether translation mode and direction had a significant impact on the distribution of participants among clusters.

Finally, to enhance interpretability and triangulate the findings, we supplemented the cluster analysis with qualitative observations derived from scanpaths and TPGs. Scanpaths and TPGs were produced by Tobii Studio and CRITT Translation Process Research Database (TPR-DB, https://sites.google.com/site/centretranslationinnovation/tpr-db), respectively.

## Results

### Effects of translation mode and direction on preparatory reading behaviors

To answer RQ1, we examined the effects of translation mode and direction on translators’ preparatory reading behaviors by analyzing three key measures: preparatory reading duration, average fixation duration, and fixation rate. The descriptive statistics of the three preparatory reading metrics are presented in Table 2.

**Table 2:** Descriptive statistics of three preparatory reading metrics.

Preparatory reading metrics	Overall	Translation mode		Translation direction	
		Sight translation (SiT)	Written translation (WT)	L1-to-L2	L2-to-L1
Preparatory reading duration (seconds)	110.483 (111.473)	170.823 (129.689)	56.279 (48.846)	108.233 (127.182)	112.577 (95.649)
Average fixation duration (milliseconds)	247.882 (37.599)	257.922 (39.046)	238.547 (33.939)	253.143 (43.415)	243.166 (31.129)
Fixation rate (count/second)	2.870 (0.523)	2.783 (0.478)	2.951 (0.553)	2.705 (0.546)	3.018 (0.457)

Notes: 1. Descriptive statistics were presented in the form of mean values, with standard deviations provided in parentheses.

2. L1-to-L2 refers to Chinese-to-English translation while L2-to-L1 refers to English-to-Chinese translation in this study.

**Fig 2:**
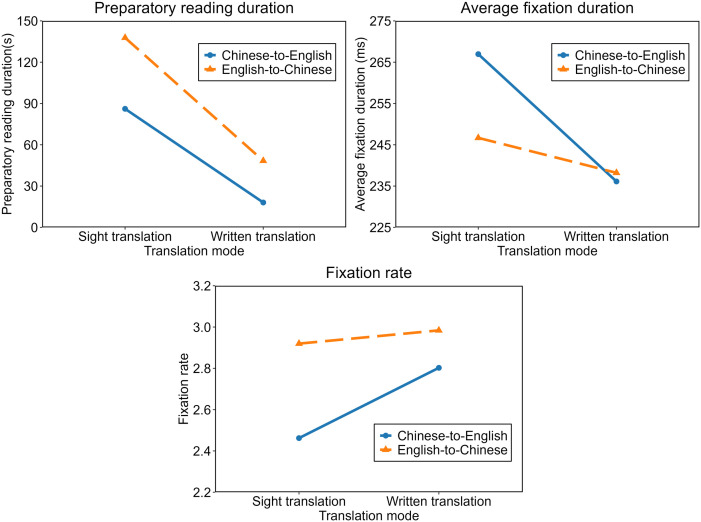
Linear mixed-effect model results.

We also conducted LMM analyses on the three measures, and the results are illustrated in Fig 2. Regarding preparatory reading duration, results showed that the main effect of translation mode was significant (*F*_*(1, 41.69)*_ *=* 141.344, **p <* *0.001): SiT preparatory reading duration was longer than WT preparatory reading (*t =* 11.866, *β =* 1.300, *SE = *0.110, *p <* 0.001). However, the main effect of translation direction was not significant (*F*_*(1, 28.28)*_ *=* 2.551, *p = *0.121), but there was a significant interaction effect between translation mode and translation direction (*F*_*(1, 246.95)*_ *=* 5.587, *p =* 0.022). *Post hoc* analyses indicated that, within the SiT mode, the preparatory reading duration did not differ significantly between the two directions (*t =* −0.996, *β =* −0.470, *SE = *0.472, *p =* 0.327), whereas in the WT mode, L1-to-L2 direction involved shorter preparatory reading duration than the opposite direction (*t =* −2.114, *β =* −0.986, *SE = *0.466, *p =* 0.043).

Regarding average fixation duration, LMM modeling revealed that the main effects of translation mode (*F*_*(1, 77.54)*_ *=* 31.767, *p <* 0.001) and translation direction (*F*_*(1, 76.36)*_ *=* 6.637, *p =* 0.012), as well as their interaction effect (*F*_*(1, 76.10)*_ *=* 10.524, *p =* 0.002) were all statistically significant. SiT preparatory reading was associated with a longer average fixation duration than that WT, but this achieved statistical significance only in L1-to-L2 translation direction (L1-to-L2: *t =* 6.096, *β =* 0.123, *SE = *0.020, *p <* 0.001; L2-to-L1: *t =* 1.858, *β =* 0.035, *SE = *0.019, *p =* 0.067). Besides, L1-to-L2 direction involved longer average fixation duration than L2-to-L1 direction; however, this was significant only in SiT (*t =* 4.039, *β =* 0.079, *SE = *0.020, *p <* 0.001), but not in WT (*t =* −0.469, *β =* −0.009, *SE = *0.019, *p =* 0.641).

Regarding fixation rate, LMM results showed that the main effects of translation mode (*F*_*(1, 78.93)*_ *=* 8.722, *p = *0.004), translation direction (*F*_*(1, 76.37)*_ *=* 21.630, *p <* 0.001), and their interaction effects (*F*_*(1, 75.81)*_ *=* 4.665, *p =* 0.034) were all significant. *Post hoc* analyses revealed that fixation rate of preparatory reading in SiT was lower than WT, but this was significant only in L1-to-L2 direction (*t =* −3.503, *β =* −0.130, *SE = *0.037, *p =* 0.001), and not in L2-to-L1 direction (*t =* −0.629, *β =* −0.022, *SE = *0.035, *p =* 0.531). Moreover, L1-to-L2 direction was found to involve a lower fixation rate compared to the opposite direction, but this achieved significance only in SiT (*t =* −4.731, *β =* −0.171, *SE = *0.036, *p <* 0.001), not in WT (*t =* −1.806, *β =* −0.063, *SE = *0.035, *p =* 0.075).

### Preparatory reading patterns

**Fig 3:**
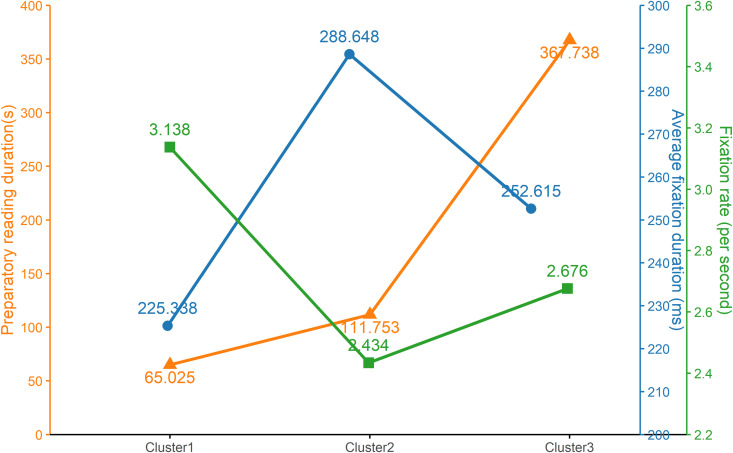
Visualizations of three clusters.

RQ2 combines both quantitative and qualitative analyses to identify distinct preparatory reading patterns. We initially utilized *k*-means cluster analysis to automatically identify global behavior patterns in preparatory reading, resulting in three distinct preparatory reading clusters. As depicted in Fig 3, the three clusters differed significantly in preparatory reading duration (*F =* 116, **p <* *0.001), average fixation duration (*F =* 72.17, *p <* 0.001), and fixation rate (*F =* 33.65, *p <* 0.001). Further pairwise comparisons were conducted and all pairwise comparisons reached statistical significance, except the fixation rate comparison between Cluster 2 and Cluster 3. To gain deeper insights into specific characteristics of each cluster, the first and second authors coded and categorized the scanpath visualizations from Tobii Studio and translation progression graphs (TPGs) of each task’s preparatory reading. The coding scheme was developed by integrating the foundational framework consisting of head-starters, quick-planners, scanners, and systematic-planners [3,33] with additional modifications derived from our initial empirical data analysis (see Appendix 1 in the S1 File & S2 File for details). The inter-coder agreement was 86.24%, with discrepancies resolved through discussion.

Each cluster represented a distinct preparatory reading approach. As shown in Table 3, Cluster 1 emerged as the most predominant approach, accounting for more than half of the total frequency (*n =* 64, 58.2%). This approach was characterized by the shortest preparatory reading duration (*M =* 60.025, *SD =* 45.052), the shortest average fixation duration (*M =* 225.338, *SD = *23.445), and the highest fixation rate (*M =* 3.138, *SD = *0.398), relative to the other clusters. When engaging in preparatory reading in this style, translators typically spent the least time and the least cognitive efforts in pre-reading the source text, doing so at the fastest speed. Results from scanpaths and TPGs (see Appendix 2 in the S1 file) further revealed that Cluster 1 predominantly involved patterns characterized by reading the source text only once or partially (*n =* 45, 70.3%). Within this cluster, a large proportion of preparatory reading featured strategies of scanning or quick-planning (*n =* 43, 67.2%). This suggests that preparatory reading of this approach likely involves making quick preparations by scanning the entire source text rapidly or, in some cases, not reading through it fully.

**Table 3:** Summary of three preparatory reading clusters.

Clusters	Preparatory reading duration (second)		Average fixation duration (millisecond)		Fixation rate (count/second)	
	Mean	*SD*	Mean	*SD*	Mean	*SD*
Cluster 1 (*n =* 64, 58.2%)	65.025	45.052	225.338	23.445	3.138	0.398
Cluster 2 (*n =* 34, 30.9%)	111.753	70.118	288.648	23.429	2.434	0.394
Cluster 3 (*n =* 12, 10.9%)	367.738	112.132	252.615	33.101	2.676	0.541

Notes: *SD* = standard deviation. Cluster 1 = *Fast Surface-level Preparatory Reading;* Cluster 2* = *Systematic Deep-level Preparatory Reading;** Cluster 3 *= *Extended Iterative Preparatory Reading.**

Cluster 2, the second most predominant cluster, consisted of 30.9% of the total (*n =* 34). This cluster was characterized by a preparatory reading duration that was of short-to-medium length (*M =* 111.753 seconds, *SD =* 70.118), the longest average fixation duration (*M =* 288.648 ms, *SD =* 23.429), and the lowest fixation rate (*M =* 2.434 fixations per second, *SD =* 0.394). These attributes indicate that such preparatory reading involves translators investing, comparatively, the highest cognitive effort and reading at the slowest speed, while still spending a moderate amount of preparation time. Analyses of scanpaths and TPGs (See Appendix 3 in S1 File) further revealed that Cluster 2 was largely characterized by a single complete reading of the source text (*n* = 20, 60.6%), with a strong tendency to revisit or closely examine certain sections (*n =* 29, 87.9%). In other words, translators in this cluster typically pre-read the source text once, but frequently revisit particular parts or conduct close reading of certain sections.

Cluster 3 emerged as the least prevalent approach (*n =* 12, 10.9%). This cluster was associated with the longest preparatory reading duration (*M =* 367.738 seconds, *SD =* 112.132), a medium level of average fixation duration (*M =* 252.615 ms, *SD =* 33.101), and a moderate fixation rate (*M =* 2.676 fixations per second, *SD =* 0.541). These characteristics mean that translators generally spent extensive time on preparatory reading, engaging in a moderate level of cognitive effort and maintaining a moderate reading speed. Analyses of scanpaths and TPGs (See Appendix 4 in the S1 File) further revealed that Cluster 3 was featured by multiple times of source text reading (*n =* 9, 75.0%). Another notable feature was that all tasks featured systematic reading of the source text, with no cases of scanning or quick planning.

We further analyzed the distributions of the three clusters across the two modes and directions to explore the effects of translation mode and translation direction. Within Cluster 1, two modes showed significant difference, with a higher frequency in WT than in SiT (41 vs. 23, *p =* 0.011). However, the distributions across two directions showed no significant difference, although L2-to-L1 direction involved a higher occurrence than L1-to-L2 direction (38 vs. 26, *p =* 0.102). Within Cluster 2, there was a relatively even distribution across two translation modes (18 vs. 16, *p =* 0.852) and two translation directions (21 vs*.* 13, *p =* 0.191), with no salient cross-modal or cross-directional differences observed. Notably, Cluster 3 involved exclusively the SiT mode, with no occurrence of WT tasks (12 vs. 0, p < 0.001), while its distribution across two directions showed no notable difference (5 vs. 7, *p =* 0.768).

## Discussions

In this study, we investigated preparatory reading behaviors in bidirectional SiT and WT, with a special focus on how translation mode and translation direction influence preparatory reading patterns. We examined three key metrics, namely preparatory reading duration, average fixation duration and fixation rate.

### How translation mode and direction influence preparatory reading behaviors

To answer RQ1, i.e. to what extent translation mode and direction modulate translators’ preparatory reading behaviors, our findings showed that SiT involved longer preparatory reading duration, longer average fixation duration, and lower fixation rate compared to WT. This suggests that SiT preparatory reading requires greater cognitive effort and attention, consistent with prior research [32]. Moreover, our phase-level analysis extends Jiménez Ivars’ [24] findings on SiT’s higher cognitive demands by offering a finer-grained account of how this manifests during the preparatory reading stage.

The heightened cognitive effort and increased attention observed during SiT preparatory reading may stem from fundamental differences in how SiT and WT constrain the subsequent drafting or re-expression phase. As a hybrid of translation and interpreting [14], SiT imposes constraints akin to those in interpreting: translators are expected to produce fluent oral renditions, typically in a single pass and under time pressure [20]. This demand for immediate, fluent output affords minimal opportunities for revision or reflection during delivery. Consequently, to effectively meet these challenges, translators usually invest greater cognitive resources during the preparatory reading phase, not only to comprehend the source text but also to mentally rehearse the forthcoming translation and anticipate potential translation challenges (see Quotes 1 & 2). In contrast, WT allows for a more flexible and iterative workflow, allowing translators to identify and resolve problems progressively through repeated reading and ongoing revision [35]. This flexibility reduces the need for extensive preparation (see Quote 2). This interpretation is supported by qualitative data from the post-experiment interviews with the translators. For example,

Quote 1: With limited chance for revisions, sight translation makes me pre-read the source text carefully and I even rehearse the translation in my mind during preparatory reading. In contrast, written translation allows for flexible revisions throughout the subsequent translation process. (Translator 07)

Quote 2: In sight translation, there is limited time for on-the-spot thinking, so I allocate more time to preparation beforehand. In contrast, during written translation, I can revise and refine while typing, which reduces the need for extensive preparatory reading. (Translator 28)

However, interpreting this result necessitates consideration of participants’ self-reported translation proficiency levels. Participants in this study reported lower proficiency in SiT compared to WT. While we argue that the increased preparatory effort primarily reflects the inherent demands of SiT, it is plausible that lower proficiency in SiT also contributed to the heightened cognitive load and attentional investment observed during the preparatory phase. This aligns with previous findings linking lower proficiency to increased processing demands [30,31], which may prompt compensatory strategies, such as more intensive preparation, particularly in less familiar or more challenging modalities. Therefore, while task-related demands are clearly central, this possible influence of proficiency underscores the need for future studies with participants equally proficient in both SiT and WT, to better disentangle task-specific effects.

Regarding the modulating effect of translation direction, our findings indicate that its influence varies across translation modes. In SiT, preparatory reading in the L1-to-L2 direction involved significantly greater cognitive effort and slower reading, as evidenced by longer average fixation durations and lower fixation rates compared to the L2-to-L1 direction. This likely reflects the well-established asymmetry in translation difficulty [28], where producing target language output in L2 typically imposes greater processing demands than translating into one’s L1. This asymmetry is especially pronounced in SiT, likely due to its inherent demands for fluent oral delivery with little chance for revision. Consequently, translators are compelled to anticipate and plan for L2 production challenges earlier, leading to more intensive preparatory reading. This interpretation is consistent with Su and Li [12], who also observed elevated cognitive load during L1-to-L2 SiT preparation, likely driven by the need to manage anticipated production challenges.

In WT, we observed significantly longer overall preparatory reading durations in the L2-to-L1 WT, indicating greater attention allocation before translation onset. This finding directly supports the results of Qassem and Al Thowaini [5], who reported a median preparatory reading duration of 138 seconds for L2-to-L1 WT, compared to only 43 seconds for L1-to-L2 in the English-Arabic language pair. It also resonates with the finding of Feng [28], who, within the English-Chinese context, found that L2-to-L1 WT involved more systematic planning, while L1-to-L2 was marked by more superficial scanning. Given that re-expressing effort can be deferred to the drafting phase in WT, this extended preparatory phase likely reflects greater cognitive investment in comprehending the L2 source text, which typically requires more processing time than the L1.

Overall, these observed directional differences in preparatory reading likely reflect translators’ perceived translation difficulty (see Quote 3) and their subjective assessments of L2 processing demands—some emphasizing comprehension difficulties (see Quote 5), others focusing on production challenges (see Quote 4).

Quote 3: My proficiency in English-to-Chinese translation is somewhat higher than in Chinese-to-English translation. Translating from one’s native language into a foreign language is generally more challenging, so I make more preparations. (Translator 05)

Quote 4: For English-to-Chinese translation, I focus on understanding the English source text, believing that expressing it in Chinese will be straightforward. In contrast, for Chinese-to-English translation, I scrutinize each word individually to ensure accurate expression in later sight translation. (Translator 14)

Quote 5: I (pre)read source text more carefully when translating from English to Chinese, whereas I read source text less carefully when translating from Chinese to English. Since English is not my native language, understanding it takes a bit more time. (Translator 10)

Furthermore, a significant interaction between translation direction and mode was observed across all three metrics. Cross-directional differences were more pronounced in SiT than in WT, while mode-based variations were greater in L1-to-L2 translation, except for total preparatory reading time. These patterns suggest that direction and mode do not operate independently but jointly shape preparatory reading behaviors. Rather than responding to each factor in isolation, translators appear to engage in integrated, context-sensitive adjustments, allocating cognitive resources in response to the combined demands of target language processing, source text comprehension, and situational constraints such as time pressure or delivery fluency. This underscores their capacity for flexible, demand-adaptive nature of preparatory reading behavior. By foregrounding the interaction between translation direction and mode, this study extends prior work that has typically examined these variables in isolation [5,12,28,32], and contributes to a more nuanced account of the cognitive mechanisms underlying preparatory reading in translation.

### Preparatory reading patterns

To answer the second research question (RQ2), we integrated the three metrics to explore translators’ preparatory reading patterns and examined how these patterns are modulated by translation mode and direction. The *k*-means cluster analysis revealed three distinct clusters of preparatory reading behaviors, each characterized by a unique configuration of preparatory reading duration, average fixation duration, and fixation rate.

The first pattern, emerging as the most prevalent, involved translators dedicating the least amount of attention and effort while reading at the fastest speed. Scanpaths and TPGs revealed that preparatory reading in this cluster primarily involved scanning or quick planning [2,3,28], with translators swiftly scanning the entire source text just once or reading only a few words or sentences. Another notable feature lies in its average fixation duration (*M =* 225 ms), which closely mirrors the mean fixation duration typically observed during silent reading for comprehension [55]. This similarity led us to infer that the cognitive effort involved in this preparatory reading style resembles that of silent reading for general understanding. It is likely that the purpose of such preparatory reading is to achieve a general comprehension of the source text, rather than exhaustive pre-processing. This points to an underlying cognitive mechanism geared toward speed over depth.

Consequently, we termed this pattern *Fast Surface-level Preparatory Reading*, reflecting a cognitive strategy aimed at quick information extraction with minimal cognitive effort. Reading behaviors associated with this pattern resemble the “successive processing strategy” described by Krings [26] as well as the “macro-reading stage” proposed by Mikkelson [29]. Moreover, this pattern appeared significantly more frequently in WT, nearly twice as often as in SiT, which supports our earlier finding that WT preparatory reading entails less intensive cognitive engagement. This higher prevalence of the *Fast Surface-Level Preparatory Reading* pattern in WT suggests that it functions as a common strategy for forming a rapid, good-enough understanding of the source text without extensive cognitive investment.

Cluster 2, the second most predominant pattern, represents a more deliberate and comprehensive approach to preparatory reading. This pattern was characterized by a moderate level of attention, the highest cognitive effort, and the slowest reading speed. Analyses of scanpaths and TPGs further revealed that this cluster was largely associated with a single, systematic reading of the entire source text [2,3,28], with a strong tendency for recursive reading. This suggests a cognitive mechanism oriented toward in-depth analysis, with translators engaging in close inspection and reprocessing of the source text where necessary. Notably, the average fixation duration in this cluster was 288.648 ms, substantially longer than that of reading for comprehension [55], reflecting a relatively deeper and more meticulous engagement with the source text. This elevated cognitive load may reflect attempts to identify and address potential translation problems during the preparatory phase through recursive reading. Thus, we termed this pattern as *Systematic Deep-level Preparatory Reading*. The relatively low silhouette coefficient (0.06), however, suggests that this pattern should be interpreted with some caution, as it may reflect a degree of internal variability in preparatory reading behaviors.

This preparatory reading style resembles reading strategies and cognitive style of Krings’ [26] “pre-translation relief strategy” and Mikkelson’s [29] “micro-reading stage”. This style also reflects slower, more effortful source-text processing. In addition, we found that the frequency of this preparatory reading pattern did not differ significantly across translation modes or directions. This suggests that the strategy may be flexibly applied across contexts, especially when time constraints are minimal.

Cluster 3 emerged as the least frequent pattern. It was marked by extended preparatory reading duration, moderate cognitive effort, and a slow-to-moderate reading speed. The average preparatory reading time in this cluster was more than five times longer than that of Cluster 1 and over three times longer than Cluster 2. It also substantially exceeded the self-paced preparatory reading times reported in previous studies on both SiT and WT tasks [12,32], marking it as a distinct feature of this pattern. This prolonged duration can be attributed to the repeated and systematic rereading of the source text, often involving multiple full passes. As noted by participants themselves (Quote 6–7), the first round of reading typically served to establish a general understanding and identify potential translation challenges—similar to the *Fast Surface-level Preparatory Reading*. The second round involved mentally rehearsing the translation, particularly focusing on problematic segments—resembling the *Systematic Deep-level Preparatory Reading*. The iterative process of reading and re-reading aims to ensure thorough comprehension and internalization before producing the target text. Thus, we labeled this pattern *Extended Iterative Preparatory Reading*. This pattern supports Mikkelson’s [29] macro-micro sequential model of SiT preparatory reading, while refining it by highlighting the role of prospective mental rehearsal in the second stage.

Quote 6: During sight translation, I typically pre-read the source text twice. In the first reading, I identify unfamiliar phrases and sentences, and in the second, I formulate the translations in my mind. This approach enables me to generate target texts more fluently. (Translator 01)

Quote 7: During sight translation, I first read through the text to understand it. After grasping the meaning, I then ponder how to express the key phrases in the target language. (Translator 28)

Notably, this *Extended Iterative Preparatory Reading* approach was exclusively observed in SiT tasks, reinforcing the idea that SiT demands a more extensive preparatory reading process. The extended and iterative nature of this preparatory reading pattern appeared particularly well-suited to SiT, where sufficient preparation—known to ease perceived interpreting difficulty [23]—helps translators feel more confident in achieving fluent and accurate oral output.

### Pedagogical implications

The above findings and discussions underscore the importance of developing differentiated training approaches tailored to translators’ preparatory reading profiles. For translators exhibiting *Fast Surface-level Preparatory Reading*, instruction should prioritize strengthening source-text analytical skills—particularly the ability to quickly extract main ideas, trace logical progression, and infer implicit meaning beyond surface-level comprehension. This can be supported through timed, focused tasks—such as one-minute summarization, logical mapping of argument flow, or scanning for key content—which promote quick yet cognitively engaged reading under time constraints. Such activities are well suited to the preparatory phase of SiT and WT training, where time-efficient comprehension is essential.

Translators displaying *Systematic Deep-level Preparatory Reading* and *Extended Iterative Preparatory Reading* would benefit from interventions aimed at improving processing efficiency while preserving essential depth of comprehension. To achieve this, training can focus on improving the efficiency with which translators handle common problem areas—such as idioms, ambiguous referents, or complex syntax—under time constraints. Useful practice activities include chunking tasks that train learners to segment the text into meaningful units, and pre-translation annotation to identify and prioritize cognitively demanding segments. Comparing outcomes from different reading strategies can also help students develop selective depth and improve decision-making under pressure. Such individualized training fosters strategic flexibility, enabling translators to dynamically adjust their reading approaches in response to the varied cognitive demands inherent in professional translation contexts.

## Conclusions

This study explored translators’ preparatory reading behaviors in bidirectional sight translation (SiT) and written translation (WT) using eye-tracking. Three behavioral metrics of preparatory reading—preparatory reading duration, average fixation duration, and fixation rate—were analyzed to identify preparatory reading patterns. Overall, we found that translation mode played a primary role in determining the nature and depth of preparatory reading, with SiT consistently requiring more cognitive effort and attentional resources than WT, as evidenced by significantly longer total reading durations and higher average fixation durations. Translation direction further modulates these effects in intricate ways, adding complexity to how preparatory reading behaviors are shaped. By applying cluster analysis to the three metrics, we identified three distinct preparatory reading styles—*Fast Surface-Level Preparatory Reading, Systematic Deep-Level Preparatory Reading*, and *Extended Iterative Preparatory Reading*—thereby providing new insights into existing models of preparatory reading. This study highlights the need for translator training to include reading strategies tailored to the cognitive demands of different modes and directions, as well as preparation techniques adapted to specific behavioral patterns, to improve overall translation performance.

### Limitations and future directions

Despite these contributions, this study has several limitations. One limitation is that the sample consisted solely of student translators, which may not fully represent the reading strategies used by professionals. Given their greater experience and domain knowledge, professional translators may demonstrate different cognitive patterns. Future research could therefore explore whether similar clustering patterns are observed in more experienced translator populations. Another limitation lies in our choice of eye-movement metrics that focused primarily on fixations, which may not capture specific reading behaviors such as regressions and saccadic movements. Incorporating additional eye-movement metrics, such as measures of regressions and saccadic amplitude, could provide a more comprehensive understanding of reading processes. A further limitation involves potential confounding from individual differences, such as reading speed and cognitive capacity. While we accounted for participant-level variability by including random effects in the mixed-effects models and applied data standardization, these procedures may not fully capture the complexity of individual cognitive profiles. Future research could incorporate baseline measures, such as pre-experiment reading tests, to better control for these factors. Additionally, this study did not examine the relationship between reading patterns and translation performance, primarily due to the methodological challenges in comparing quality across written and oral translation modalities. Nonetheless, we recognize the theoretical and pedagogical value of linking process data with translation outcomes. Future research could build on the present findings to address these methodological challenges and further investigate how preparatory reading strategies relate to translation quality, such as accuracy and fluency.

## Supporting information

S1 File This file contains four supplementary tables (i.e. Appendix 1–4) presenting the distribution of specific preparatory reading styles across the three identified clusters. (DOCX)

S2 File This file provides an overview of Translation Progression Graphs (TPGs) and scanpath visualizations, and includes nine sets of figures illustrating representative TPGs and scanpaths for different preparatory reading styles. (DOCX)
