# Supplementary material for: Exploring preparatory reading in bidirectional sight and written translation through clustering analysis of eye-tracking data
Source: PLoS One. 2025 Aug 26;20(8):e0329858. doi: 10.1371/journal.pone.0329858 (PMC12380311; doi:10.1371/journal.pone.0329858)
Supplement: S1 File — (DOCX) [file pone.0329858.s001.docx]

**S1 Appendix 1. Preparatory reading styles identified through translators’ scanpaths and Translation Progression Graphs (TPGs)**

| **Preparatory reading styles** | **Definitions** | **Number of times for complete reading** |
| --- | --- | --- |
| Quick planning | Rapidly reading initial segments of the source text. | < 1 |
| Quick planning with local close reading | Rapid reading of initial parts of the source text with focused, detailed reading of specific words or phrases. | ≤ 1 |
| Scanning the whole text | Quickly scanning the entire source text in one pass. | 1 |
| Scanning with local close reading | Scanning the source text broadly, combined with detailed reading of particular segments. | ≥ 1 |
| Scanning with systematic single reading | Scanning the source text broadly, combined with a thorough, linear single reading of the whole text. | > 1 |
| Systematic single reading | Reading the entire source text thoroughly and linearly once, from beginning to end. | 1 |
| Systematic single recursive reading | Reading the source text once thoroughly, often with recursive close reading of certain segments and occasional revisits to particular parts. | 1 |
| Systematic multiple reading | Reading the entire text thoroughly and linearly multiple times. | > 1 |
| Systematic multiple recursive reading | Reading the entire text thoroughly multiple times, often with recursive close reading of certain segments and occasional revisits to particular parts. | > 1 |

**S1 Appendix 2. Specific preparatory reading styles within *Fast Surface-level Preparatory Reading* approach (Cluster 1)**

| **Preparatory reading styles** | **Number of times for complete readings** | **Frequency** | **Proportion** |
| --- | --- | --- | --- |
| Scanning the whole text | 1 | 16 | 25.0% |
| Quick planning | < 1 | 14 | 21.9% |
| Scanning with local close reading | ≥ 1 | 13 | 20.3% |
| Systematic single recursive reading | 1 | 8 | 12.5% |
| Systematic single reading | 1 | 7 | 10.9% |
| Systematic multiple recursive reading | > 1 | 3 | 4.7% |
| Systematic multiple reading | > 1 | 2 | 3.1% |
| Scanning with systematic single reading | > 1 | 1 | 1.6% |
| **Total** |  | **64** | **100.0%** |

Notes:

1. Sessions with reading the source text only once or partially (*n =* 45, 70.3%) include 1) Scanning the whole text, 2) quick planning, 3) systematic single recursive reading, and 4) systematic single reading.
2. Sessions with preparatory reading featuring strategies of scanning or quick-planning (*n =* 43, 67.2%) include 1) scanning the whole text, 2) quick planning, and 3) scanning with local close reading.

**S1 Appendix 3. Specific preparatory reading styles within *Systematic Deep-level Preparatory Reading* approach (Cluster 2)**

| **Preparatory reading styles** | **Number of times for complete readings** | **Frequency** | **Proportion** |
| --- | --- | --- | --- |
| Systematic single recursive reading | 1 | 19 | 57.6% |
| Scanning with local close reading | ≥ 1 | 5 | 15.2% |
| Systematic multiple recursive reading | > 1 | 3 | 9.1% |
| Quick planning with local close reading | ≤ 1 | 2 | 6.1% |
| Quick planning | ≤ 1 | 2 | 6.1% |
| Systematic single reading | 1 | 1 | 3.0% |
| Scanning with systematic single reading | > 1 | 1 | 3.0% |
| **Total** |  | **33** | **100.0%** |

Notes:

1. Sessions with a single complete reading of the source text (*n =* 20, 60.6%) include 1) systematic single recursive reading and 2) systematic single reading.
2. Sessions with a strong tendency to revisit particular parts or do close reading of certain sections (*n =* 29, 87.9%) include1) systematic single recursive reading, 2) scanning with local close reading, 3) systematic multiple recursive reading, and 4) quick planning with local close reading.

**S1 Appendix 4. Specific preparatory reading styles *Extended Iterative Preparatory Reading* approach (within Cluster 3)**

| **Preparatory reading patterns** | **Number of times for complete readings** | **Frequency** | **Proportion** |
| --- | --- | --- | --- |
| Systematic multiple recursive reading | > 1 | 7 | 58.3% |
| Systematic single recursive reading | 1 | 3 | 25.0% |
| Systematic multiple reading | > 1 | 2 | 16.7% |
| **Total** |  | **12** | **100.0%** |

Note: Sessions featured by multiple times of source text reading (*n =* 9, 75.0%) include 1) systematic multiple recursive reading and 2) systematic multiple reading.
