# Supplementary material for: Exploring preparatory reading in bidirectional sight and written translation through clustering analysis of eye-tracking data
Source: PLoS One. 2025 Aug 26;20(8):e0329858. doi: 10.1371/journal.pone.0329858 (PMC12380311; doi:10.1371/journal.pone.0329858)
Supplement: S2 File — (DOCX) [file pone.0329858.s002.docx]

**S2 Translation progression graphs and scanpath visualizations**

Translation progression graphs (TPGs) depict how translation process evolves over time. A typical TPG depicts the whole translation process, which usually include different phases such as the orientation phase, drafting phase and revision phase in written translation (WT), or the orientation phase and re-expressing phase in sight translation (SiT). However, the following TPGs zoom in on the orientation phase of SiT and WT, focusing on how preparatory reading unfolds over time by depicting translators’ gaze patterns on the source text (ST). In TPGs, the horizontal dimension plots translators’ behavioral data in milliseconds (ms) on the X-axis. In the following TPGs, the translators’ behavioral data include only their fixation data on ST during preparatory reading. The fixations are represented by blue dots, with each dot representing one fixation. The X-axis position of each blue dot is determined by the time when the fixation occurs. The Y-axis position of each blue dot is determined by the fixation’s location, which is further mapped to the relevant ST word the fixations is on. The left Y-axis of the TPGs displays the ST and the right Y-axis of the TPGs displays the TT. The ST words are sequenced from bottom to top from the 1^st^ ST word to the last ST word. In addition, on the left Y-axis, TPGs show the segment (i.e. sentence) information, with the 1^st^ ST sentence at the bottom and the last sentence on the top. For example, in the first graph below representing 1) Quick planning, we can see from the graph that the translator was first reading linearly from ST word no. 1 to no. 40, covering the first two ST sentences (as depicted in the graph as Seg. 1 and Seg. 2), and then went back to the 1^st^ sentence to read the first few ST words.

| 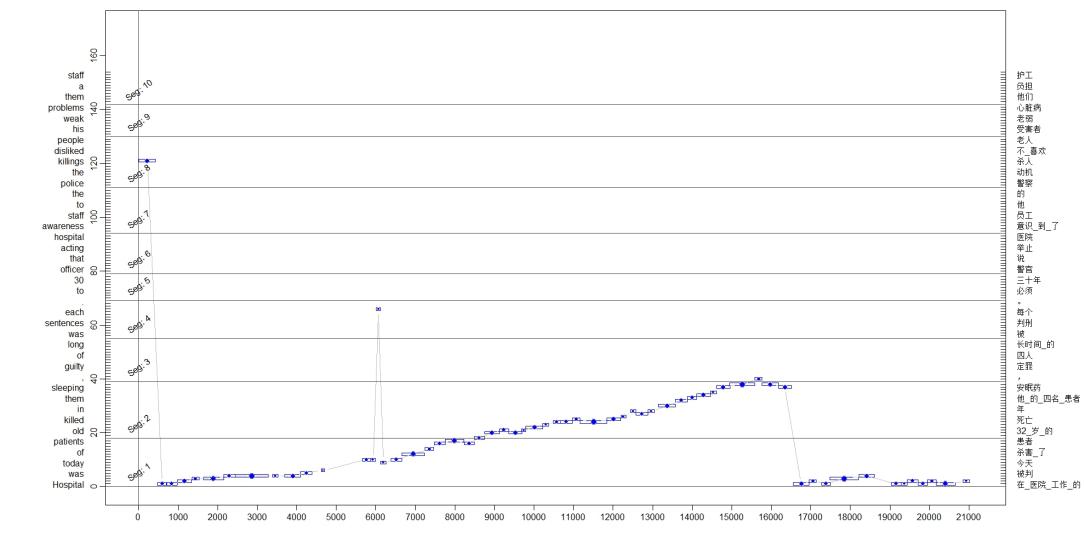 | 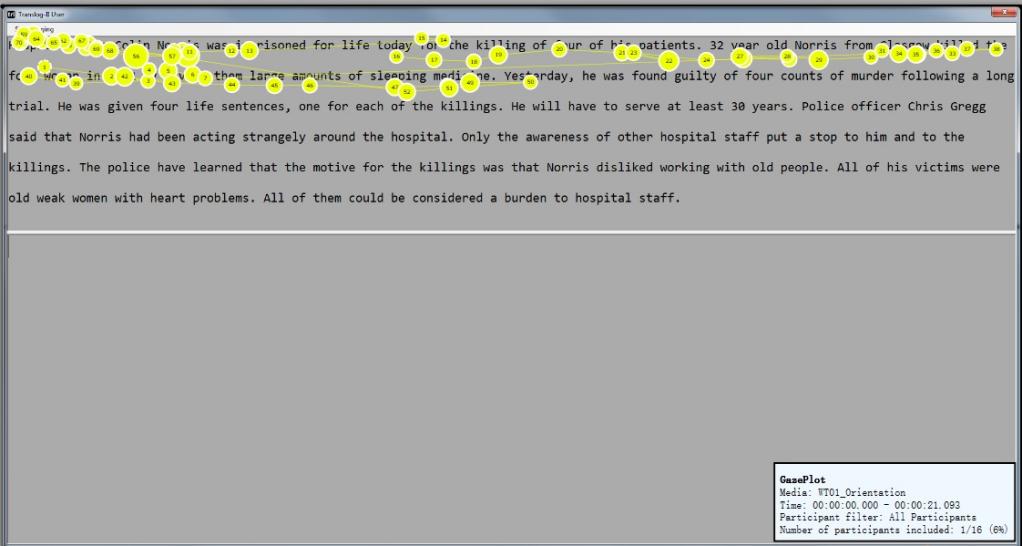 |
| --- | --- |
| Translation progression graph | Scanpath |

**S2 Fig 1. Quick planning: translation progression graph and scanpath**

Note: The translator (P12) only read the first two sentences during preparatory reading the source text (WT01).

| 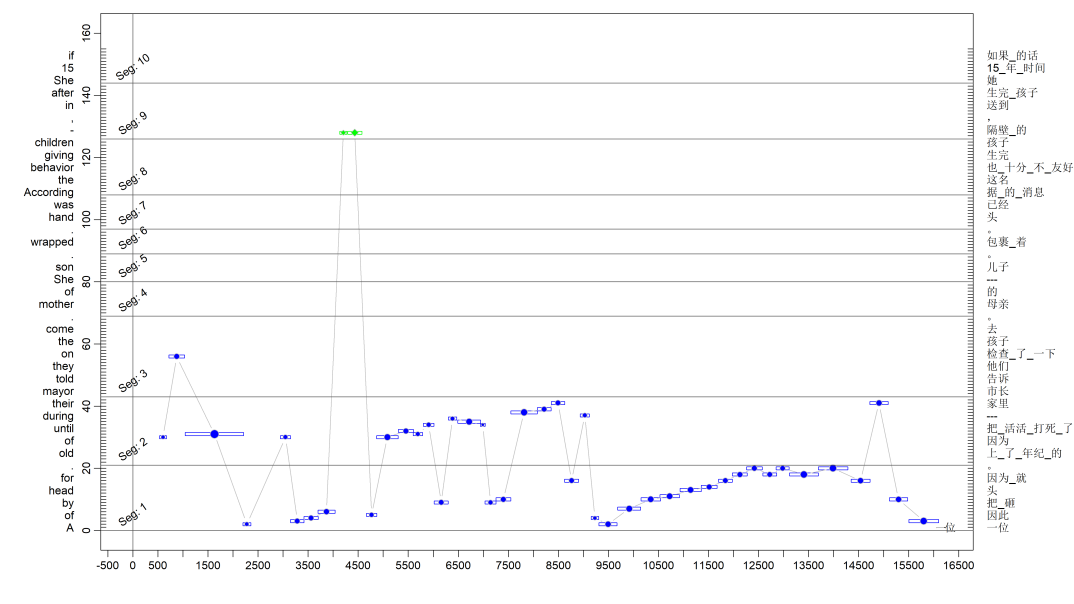 | 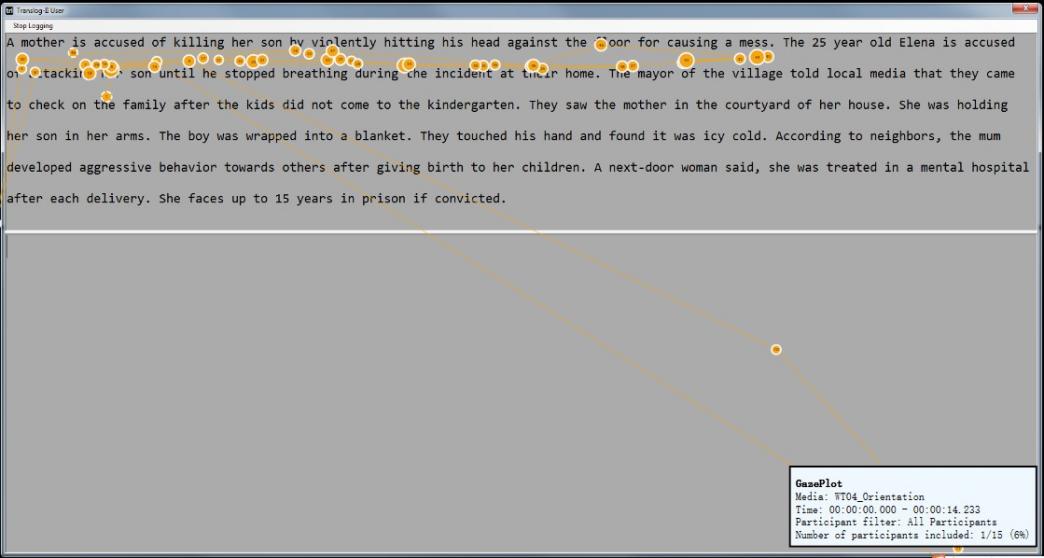 |
| --- | --- |
| Translation progression graph | Scanpath |

**S2 Fig 2. Quick planning with local close reading: translation progression graph and scanpath**

Note: The translator (P11) rapidly read the initial parts of the source text (WT04) with focused, detailed reading of specific words or phrases.

| 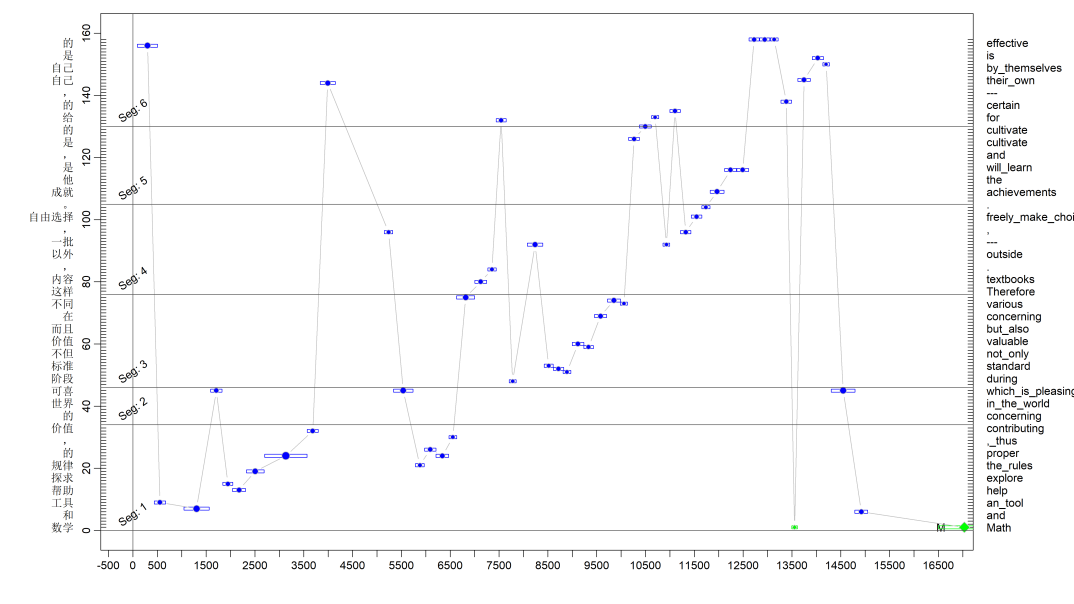 | 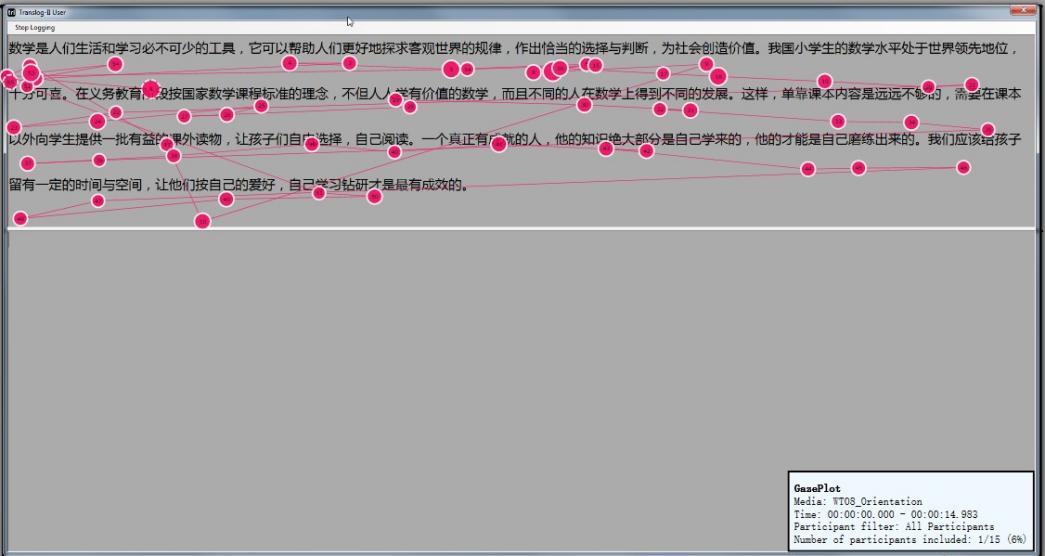 |
| --- | --- |
| Translation progression graph | Scanpath |

**S2 Fig 3. Scanning the whole text: translation progression graph and scanpath**

Note: The translator (P31) scanned the whole text (WT08) during preparatory reading.

| 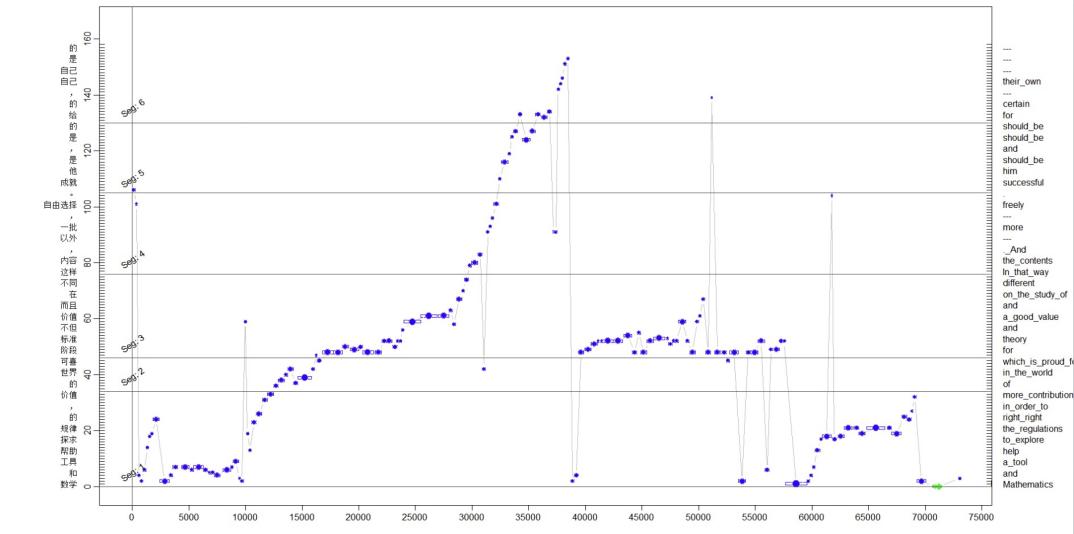 | 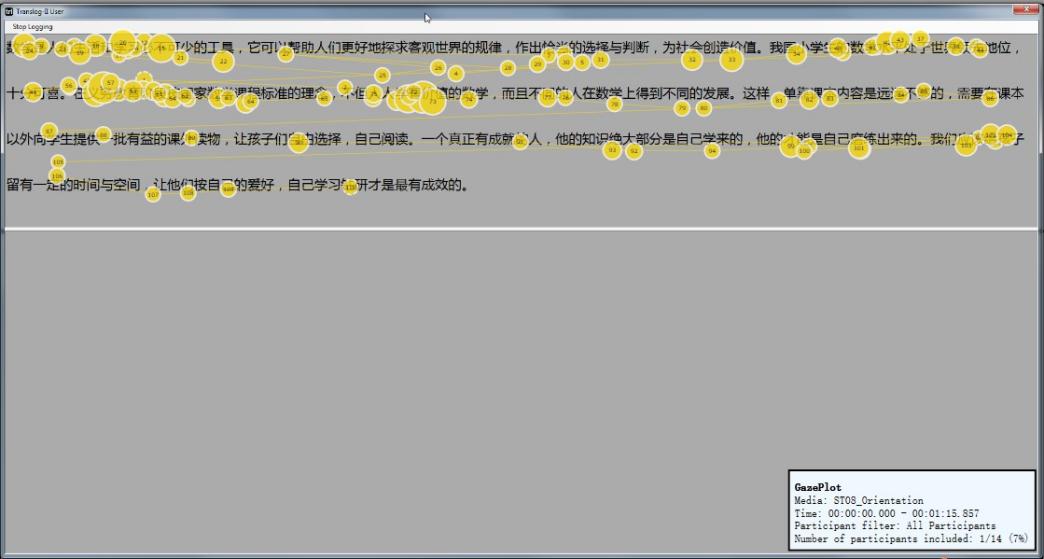  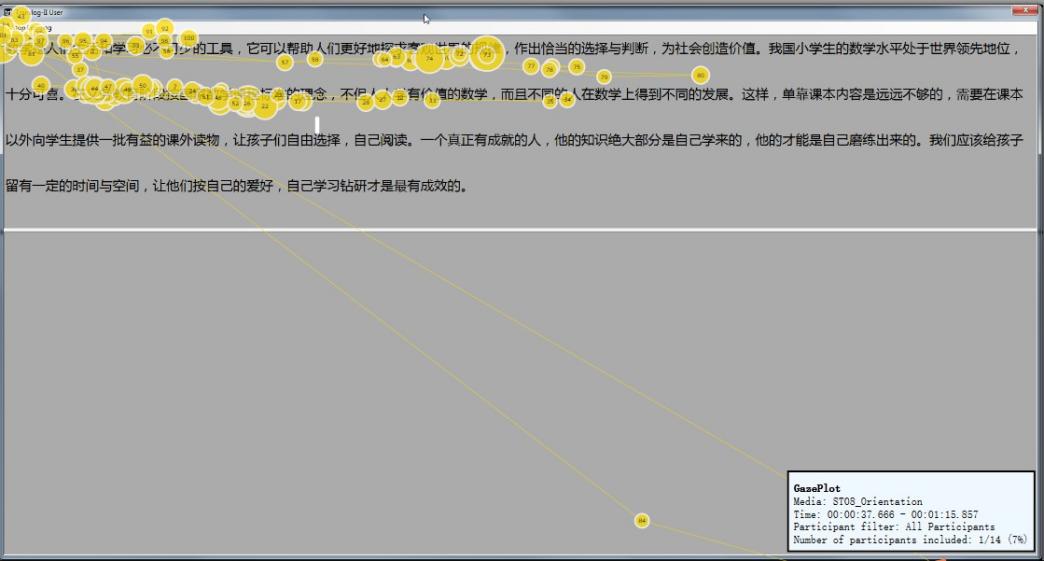 |
| --- | --- |
| Translation progression graph | Scanpath |

**S2 Fig 4. Scanning with local close reading: translation progression graph and scanpath**

Note: The translator (P24) first scanned the source text (ST08) broadly, combined with detailed reading of particular segments, i.e. the 3rd & 1st sentences in this case.

| 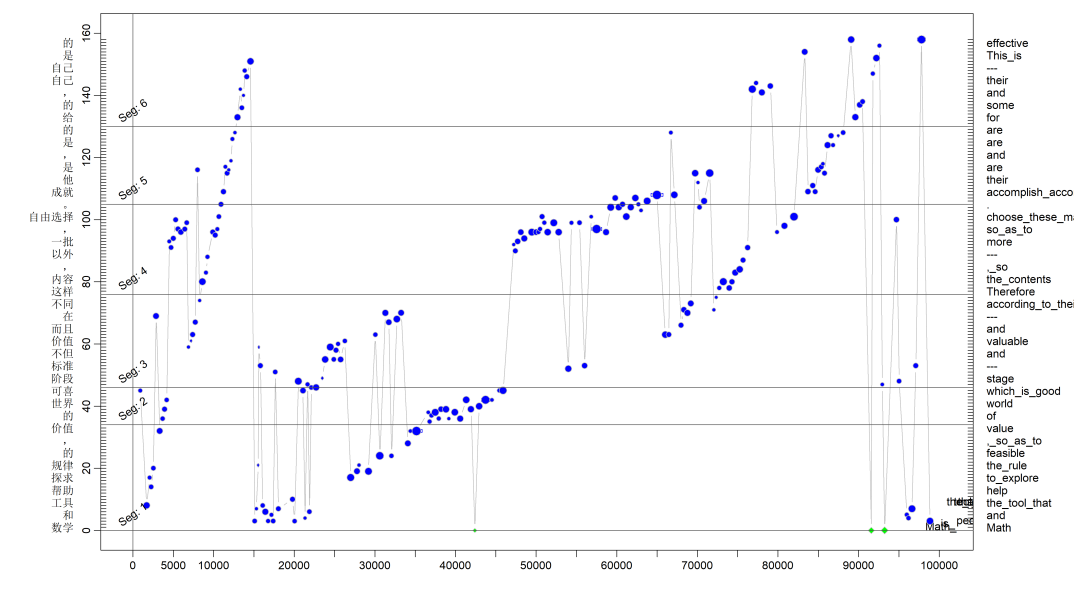 | 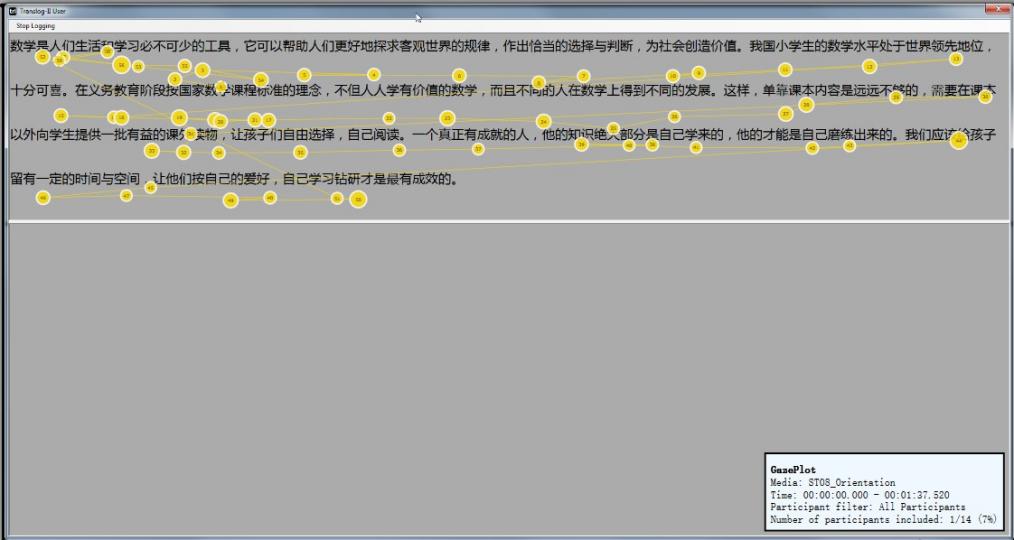  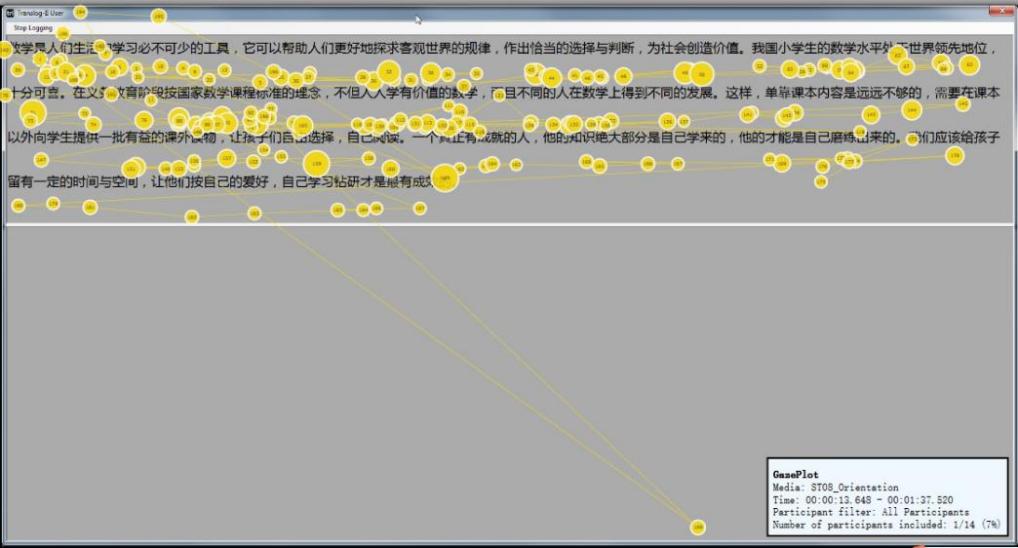 |
| --- | --- |
| Translation progression graph | Scanpath |

**S2 Fig 5. Scanning with systematic single reading: translation progression graph and scanpath**

Note: The translator (P17) first scanned the source text (ST08) broadly, then went back to the beginning to read the source text again in a systematic way.

| 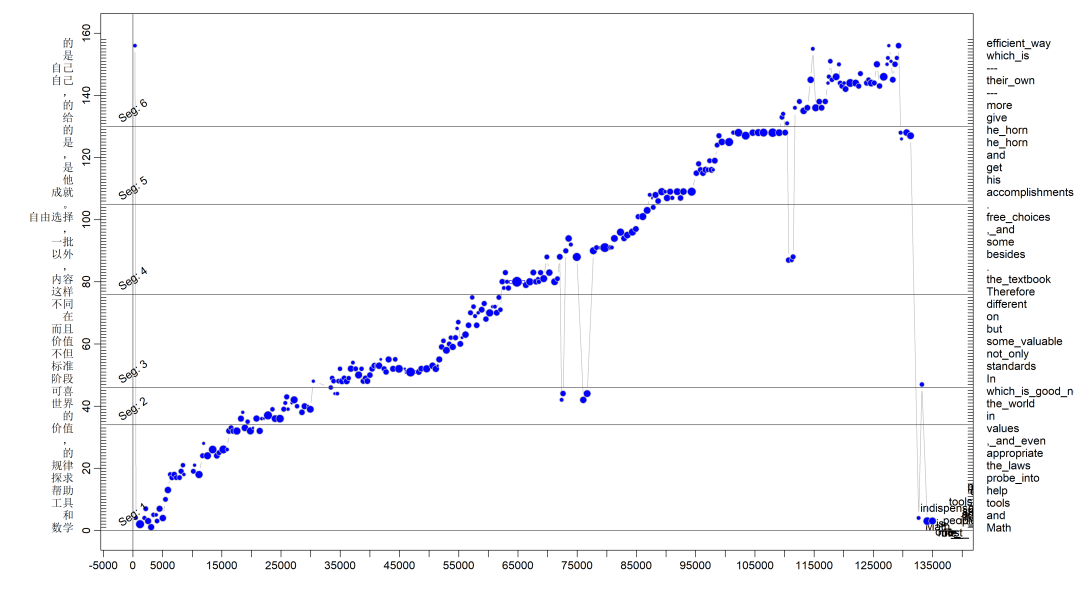 | 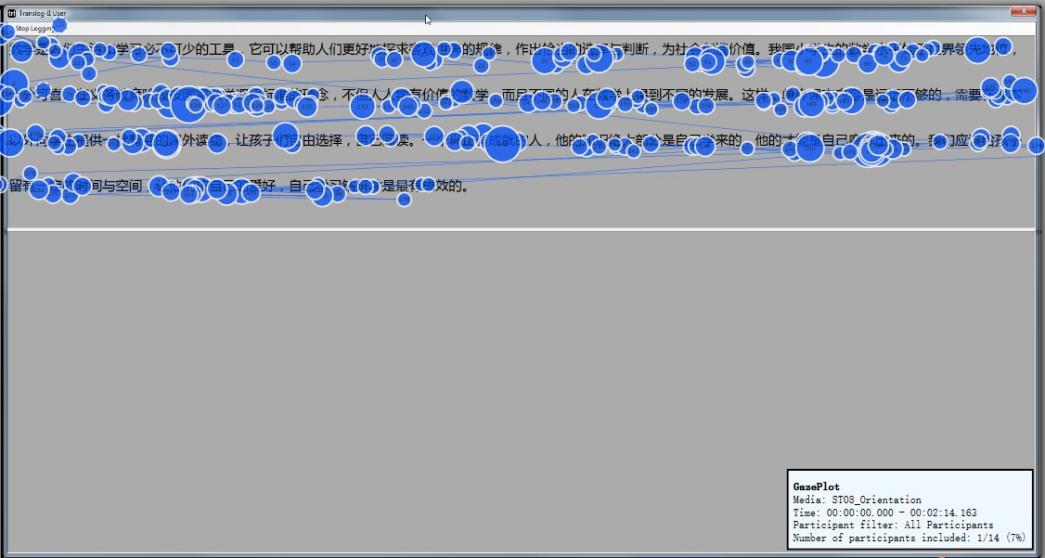 |
| --- | --- |
| Translation progression graph | Scanpath |

**S2 Fig 6. Systematic single reading: translation progression graph and scanpath**

Note: The translator (P22) read the entire source text (ST08) thoroughly and linearly once, from beginning to end.

| 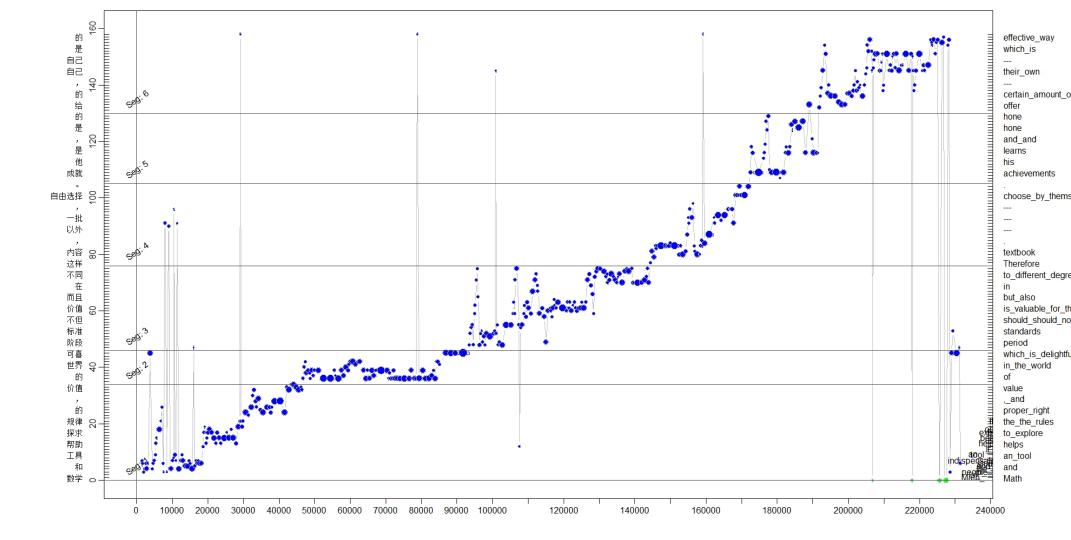 | 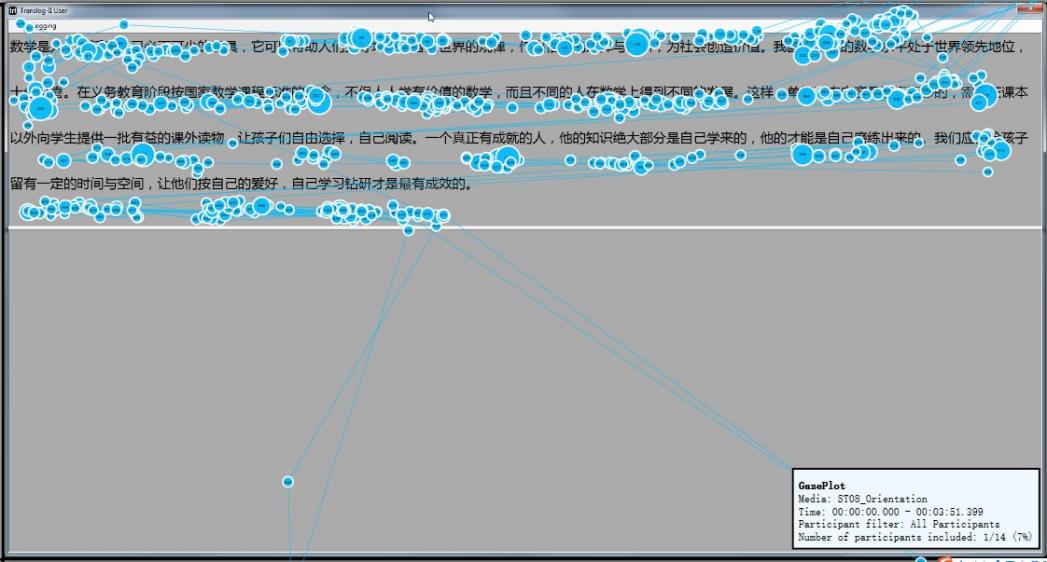 |
| --- | --- |
| Translation progression graph | Scanpath |

**S2 Fig 7. Systematic single recursive reading: translation progression graph and scanpath**

Note: The translator (P14) read the source text (ST08) once thoroughly, often with recursive close reading of certain segments and occasional revisits to particular parts.

| 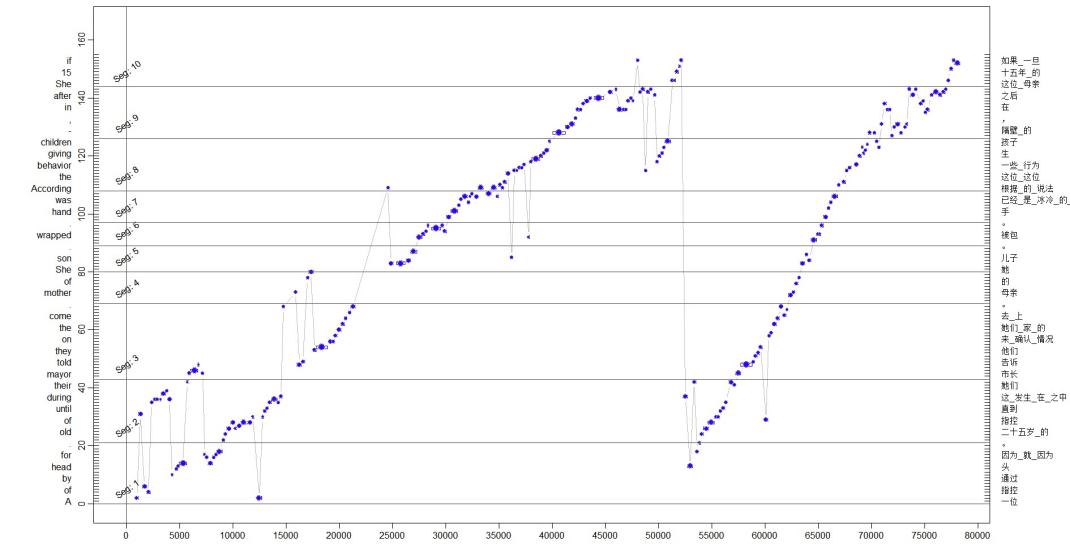 | 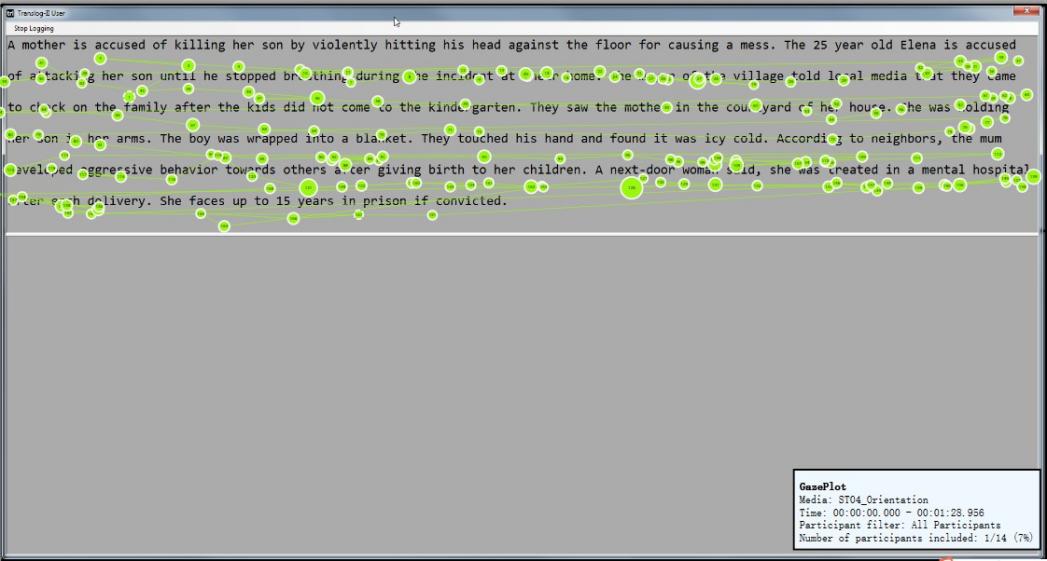  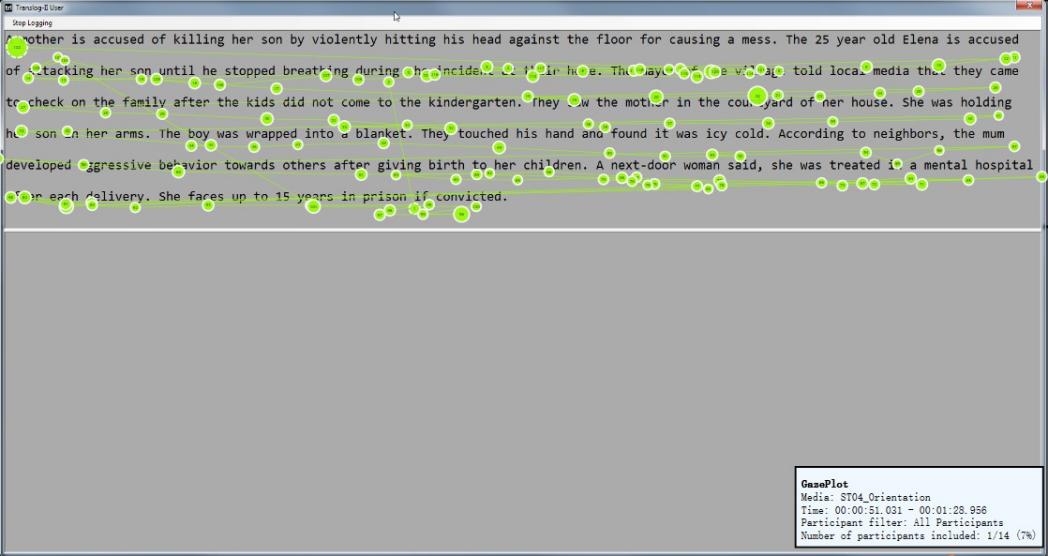 |
| --- | --- |
| Translation progression graph | Scanpath |

**S2 Fig 8. Systematic multiple reading: translation progression graph and scanpath**

Note: The translator (P13) read the entire source text (ST04) thoroughly and linearly multiple times.

| 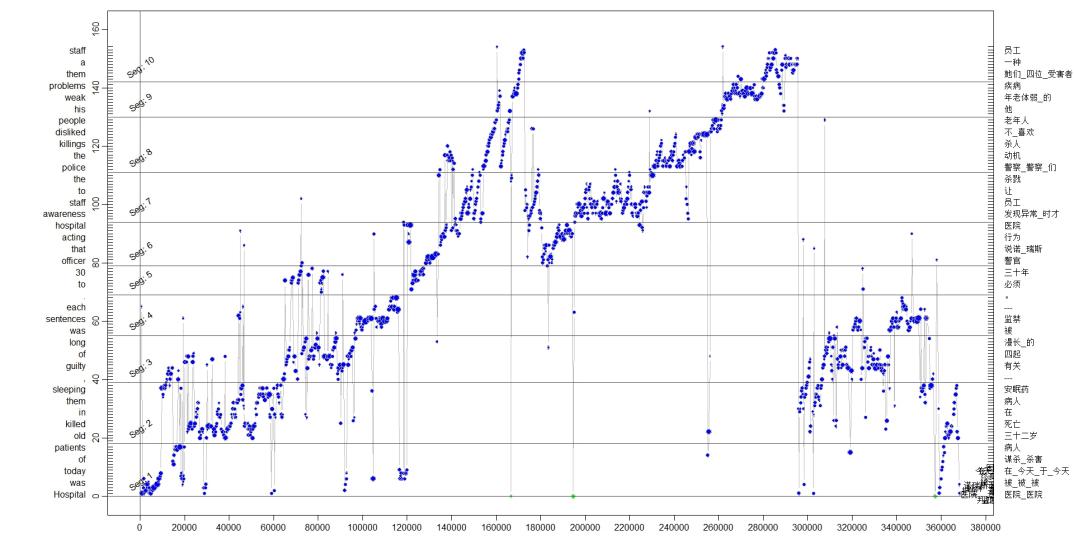 | 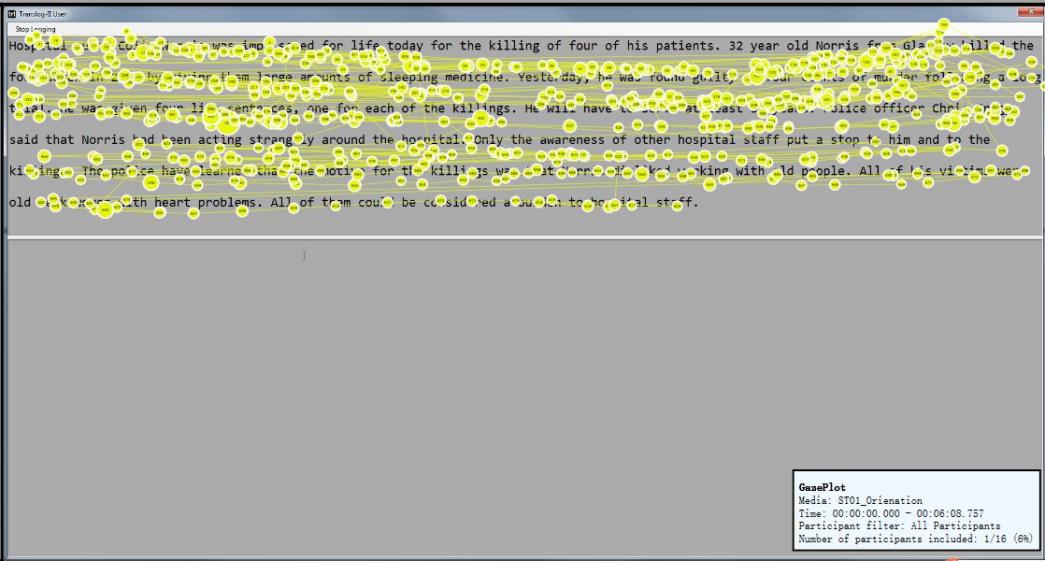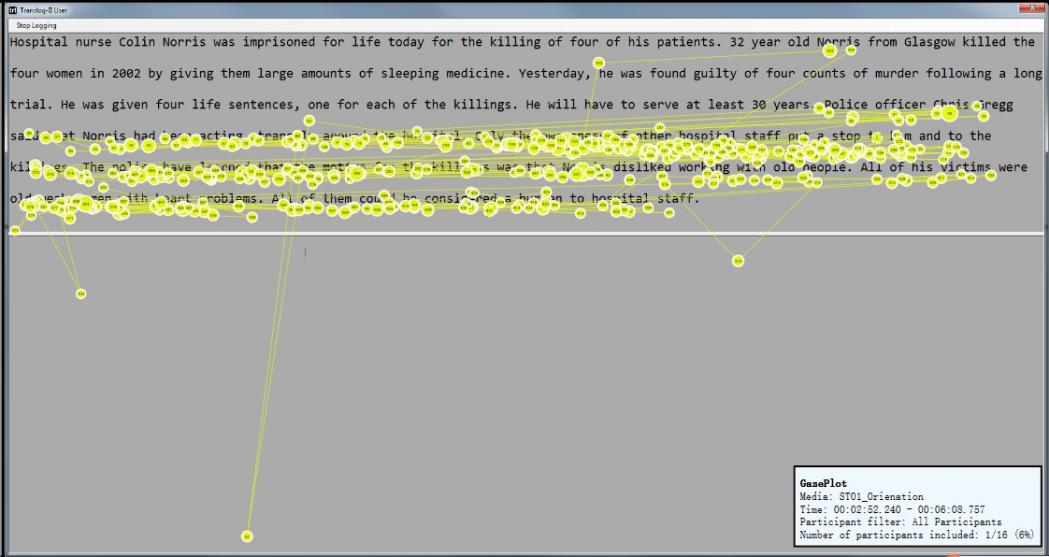  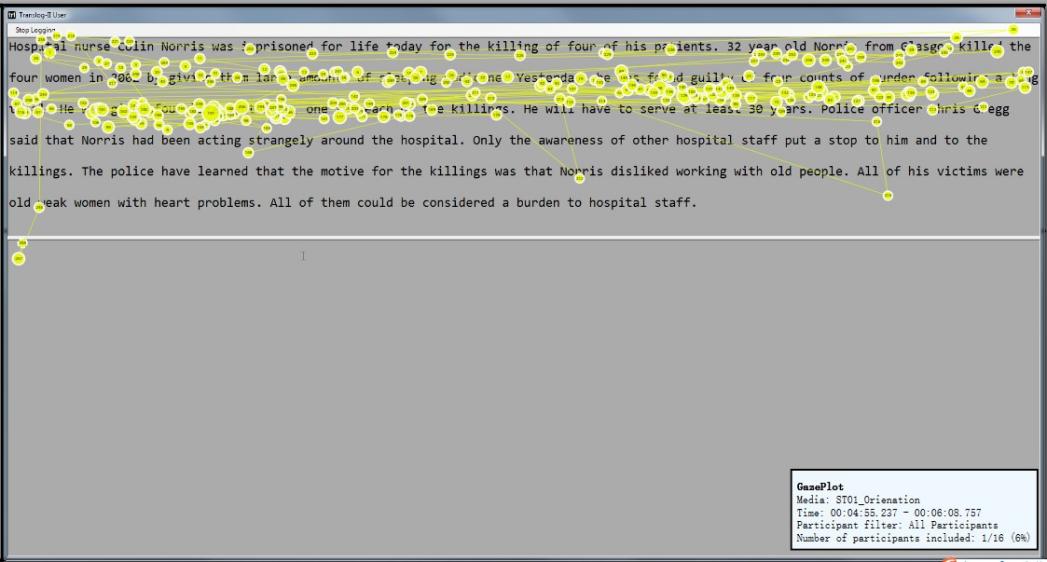 |
| --- | --- |
| Translation progression graph | Scanpath |

**S2 Fig 9. Systematic multiple recursive reading: translation progression graph and scanpath**

Note: The translator (P30) read the entire source text (ST01) thoroughly multiple times, often with recursive close reading of certain segments and occasional revisits to particular parts.
